# Offering Psychological Support to University Students in Poland During the COVID-19 Pandemic: Lessons Learned From an Initial Evaluation

**DOI:** 10.3389/fpsyg.2021.635378

**Published:** 2021-10-11

**Authors:** Agata Rudnik, Paulina Anikiej-Wiczenbach, Aleksandra Szulman-Wardal, Paul Conway, Mariola Bidzan

**Affiliations:** ^1^Institute of Psychology, University of Gdańsk, Gdańsk, Poland; ^2^Department of Gastroenterology, Independent Public Health Care of the Ministry of the Internal Affairs in Gdańsk, Gdańsk, Poland; ^3^Academic Psychological Support Center, University of Gdańsk, Gdańsk, Poland; ^4^The Specialist Hospital in Koscierzyna, Koscierzyna, Poland; ^5^Department of Psychological Sciences, Birkbeck University of London, London, United Kingdom

**Keywords:** counselling, therapeutic intervention, university students, narrative, COVID-19

## Abstract

This perspective article aims to present insights into an initiative to provide free psychological support to students at the University of Gdańsk (UG) – one of the first universities in Poland to offer such help – during the first major COVID-19 pandemic lockdown in March 2020. We begin by introducing key psychological consequences of the pandemic, with particular emphasis on university students, according to Pandemic Management Theory (PMT). Next, we highlight the most significant challenges reported to us by the students in response to the support initiative and applied psychological interventions (including psychoeducation, relaxation, arranging day plans, taking care of relationships, and “just” talking). We conclude by reflecting on the challenges for mental health posed by the COVID-19 pandemic as well as the outcome of our initiative – the creation of the Academic Psychological Support Centre at the University of Gdańsk. These insights and lessons learned from developing our practice can help enhance the effectiveness of future psychological support programs through the pandemic and beyond.

## Introduction

The local transmission phase of COVID-19 in Poland was declared to the World Health Organization (WHO) on the 10th March 2020 (World Health Organization, 2020a). On the same day, Polish government introduced measures to combat COVID-19 – closing schools and cancelling university classes and other mass events (Republic of Poland – website, 2020). In response, the Rector of the University of Gdańsk issued an order suspending all teaching, scientific, and sporting and artistic events organized at the University of Gdańsk until further notice (University of Gdańsk, 2020a, 2021).^1^ An official epidemic was declared in Poland on the 20th March 2020 by the Prime Minister, resulting in unprecedented levels of peace-time disruption to virtually all aspects of everyday life as well as associated psychological, mental, and emotional issues (Bartoszek et al., 2020; Gawrych et al., 2020; Gambin et al., 2021).

The COVID-19 pandemic is associated with numerous adverse consequences for well-being and mental health, and constitutes a new stressor, comparable to natural disasters or mass armed conflicts (Brooks et al., 2020; Castiglioni and Gaj, 2020; Fiorillo and Gorwood, 2020; Pfefferbaum and North, 2020; Venuleo et al., 2020). The pandemic created unprecedented challenges for healthcare provision in general, and counselling professionals found it difficult to adapt existing schemes or protocols to the new restrictions.

The following are the groups most frequently mentioned as being particularly vulnerable to the negative psychological consequences of the pandemic: (a) people who have direct or indirect contact with the pathogen; (b) healthcare professionals – due to exposure; (c) people who are susceptible to biological and psychological stressors, including those with mental disorders/mental health problems; and (d) people who consume a lot of news media (Fiorillo and Gorwood, 2020; Pedrosa et al., 2020; Fiorenzato et al., 2021; Wang et al., 2021). The pandemic has had many unfavorable consequences for wellbeing. Everyday living has become more stressful, resulting in psychosomatic consequences, such as sleep deprivation, sadness, emotional problems, frustration, feeling helpless, and compulsively taking precautions (Bidzan et al., 2020; Bidzan-Bluma et al., 2020; Mustafa, 2020). These consequences have been observed all over the world (Ammar et al., 2020), leading to the development of theories to better understand the negative psychological effects of the pandemic.

From the very beginning, when the epidemiological situation started to worsen, it was clear that it would affect young people in many different ways. A study on the psychological impact of the COVID-19 pandemic conducted among 7,143 college students revealed that economic effects, effects on daily life, and delays in academic activities were positively associated with levels of anxiety (Cao et al., 2020). At the same time, social support was negatively correlated with anxiety symptoms. Cao et al. (2020) suggested that the mental health of college students should be closely monitored and that effective psychological support is crucial to promoting adaptive psychological functioning during the pandemic. Researchers around the world came to similar conclusions, including Patsali et al. (2020), Son et al. (2020), Wang et al. (2020a), and Wathelet et al. (2020) who indicated an alarming number of people reporting depression, anxiety, or suicidal thoughts. Furthermore, respondents expressed fears about the pandemic’s effect on their academic work, health, and lifestyle. Therefore, it is extremely important to address mental health and provide care to all groups exposed to the psychological effects of the pandemic. It is also extremely important to find immediate solutions and put in place mental health services to minimize the psychological effects of the pandemic (Grubic et al., 2020; Liu et al., 2020; Son et al., 2020; Fruehwirth et al., 2021). As the Institute of Psychology at the University of Gdańsk, we acted as soon as possible to protect the mental health of our students and to ensure their well-being.

We based our reflections on the Pandemic Management Theory (PMT; Stueck, 2021). It is based on the biocentric health management approach and details consecutive phases of coping with the burden of the pandemic and lockdown. According to PMT, to preserve a healthy identity during the COVID-19 pandemic, it is crucial that a person maintains their connections with themselves, with others, and to nature. Thus, psychological interventions should support the maintenance of these connections or restore them if necessary (Stueck, 2021). PMT distinguishes phases of coping with the burden of the lockdown and the COVID-19 pandemic. This process is divided into an orientation phase with load interpretations, acute and chronic phases of negative load consequences, and phases with positive consequences. One of the phases distinguished in this theory is a sustainable biocentric change in ethical attitudes that is focused on the protection of the lives of others (Stueck, 2021). The support we offer made it possible to preserve people’s quality of life in the face of this unexpected threat.

## Aims and Procedures

This research project was reviewed and approved by the Ethical Committee at the Institute of Psychology at the University of Gdańsk, Poland (decision no. 30/2020). We strongly believe that ethical issues and concern for mental health are of particular importance in uncertain times, such as the COVID-19 pandemic (Townsend et al., 2020).

At the beginning of the lockdown, we spontaneously decided to provide psychological counselling in anticipation of the students’ psychological needs. Additionally, it was important to us that it be available as soon as possible, and so on the 18th March 2020, an initial formal framework was quickly developed. On the same day, information about this campaign was shared on the website of the University of Gdańsk in both Polish and English (University of Gdańsk, 2020b). An information pack was forwarded to local and national media through the Polish Press Agency as well as through relevant social media channels, including the University of Gdańsk (2020c) and Visible Hand 3City (2020; a group created to connect those needing help with those offering help). As a result, students seeking support contacted us from day one of the initiative.

Psychological support was initially provided by email (to arrange an online meeting) and *via* Skype. Support was available 24h per day. We shared duties and set up notifications as soon as someone messaged us. We could also be contacted by phone. However, we encouraged video calls to ensure we could see the person we were talking to and observe their reactions, to better assess their mental state. We should not neglect to mention that we struggled with various formalities such as efficiently creating accounts – it was a race against time to provide support as soon as possible.

Ten psychologists were involved from the outset; they co-operated with us by sharing knowledge and experience based on the types of patients they typically worked with on a daily basis. Two specialists were on permanent duty, with the remaining eight being deployed based on needs. Each of the psychologists who provided psychological support had at least 3years of experience working with adult patients (including, *inter alia*, in mental health clinics, psychiatric wards, and private practices) and/or as psychotherapists or cognitive-behavioral therapists. Specialists were assigned based on experience. For example, specialists working in crisis intervention helped those who were initially assessed as being at high risk of committing suicide, while clinical psychologists consulted for people with symptoms of mental disorders. This allowed us to provide personalized, targeted support from the outset. All the psychologists participating in the project were in regular contact with each other, sharing their experiences and providing mutual supervision.

Between the 18th March and 15th June 2020, we provided psychological support to 108 people aged between 19 and 35years (*M*=22.7; *SD*=2.8). At that time, students of various faculties and years of study contacted us (we do not want to provide more details here in order to maintain anonymity and a sense of security for people who used and currently use our services). The overwhelming majority (94%) was women, which may support the claim that women are more likely to seek help during the COVID-19 pandemic (Zhao et al., 2020). Some researchers put forward the hypothesis that women and young adults experience greater psychological distress (Qiu et al., 2020; Rossi et al., 2020). In their study, Wang et al. (2020b) showed that women have suffered a greater psychological impact due to the outbreak, as well as higher levels of anxiety, depression, and stress. The same study also revealed that students were at risk of experiencing negative psychological outcomes associated with the pandemic, for example, the potential negative impact of uncertainty around academic progression could have an adverse effect on their mental health. The psychological support initiative was recognized by both the academic community (including the Rector of the University of Gdańsk) and national media – it was publicized by the Polish Press Agency (2020), among others.

Next, we will highlight some of the most common problems that occurred during the psychological support and counselling initiative and share insights into our experience as psychologists with the aim of answering the following questions: If we were to start over with the information we have now, would we organize our activities differently? Would we change the system and the interventions used?

## Common Problems and Symptoms

The first draft of this article was written in June 2020, partly in response to a lack of clear guidelines and advice from other psychologists and psychotherapists, and constituted a record of our experiences at that time. Almost a year on from the first pandemic lockdown, we can look back on our experiences from a somewhat more distant perspective and we believe that discussing the challenges we faced is strength of our publication.

Since the first lockdown, a growing body of research has been published highlighting the impact of the pandemic and lockdowns on everyone’s mental health, and it is important to identify the needs of individual populations and social groups (Bidzan et al., 2020; Bidzan-Bluma et al., 2020; Dymecka et al., 2021a,b; Schou-Bredal et al., 2021). The students we supported as part of the initiative struggled with an extensive and diverse range of problems and challenges; these are shown in Table 1.

**Table 1:** Challenges reported by supported students.

Mental health					
In general	In specific groups				
	Pregnant students	Students with chronic diseases		Students with addictions	
Worsening of symptoms Discontinuation of therapy Limited access to medical care Fear of being affected by COVID-19 Negative emotions, higher level of anxiety Loneliness Suicidal thoughts Exacerbation of mental health symptoms	Endangered pregnancy No option for family delivery Higher anxiety, because of limited access to medical care Living apart from the child’s father	Higher anxiety because of the risk of being affected and because of limited access to medical care, or to equipment (e.g., blood tests and ultrasound) Dependence on others (e.g., shopping, delivery of medicines, and lack of support)		Increased risk of addiction relapse Rehab without assistance Limited access to free therapy	
Relationships					
Partner	Children	Parents and family members		Friends	
Conflicts with partner Deterioration of intimacy with and desire for partner Lack of privacy, space and opportunities to be alone Yearning for one’s partner, if not physically together during lockdown Fear of being infected by and/or infecting partner	Burden of constant care Inability to focus on education or work during childcare Desire to organize the day for children in a stimulating way Fear of being infected by and/or infecting one’s child Difficulty explaining the situation to the child	Conflicts with family members Lack of space to oneself Care of elder and chronically ill members of the family Fear of being infected by and/or infecting other members of the family		Lack of physical group meetings Limitation of places where you can meet Loneliness	
Daily life					
Education	Work	Schedule	Mass media	Fulfilling needs	Distance
Sense of losing experiences through remote learning Feeling less prepared for professional life Lack of motivation to study remotely	Childcare during time of remote work Greater technical skills necessary for effective remote working No access to appropriate computer equipment Low internet coverage in the countryside Financial problems Loss of work Fear of loss of work	Loss of safety due to lack of routines Low motivation Feeling of inefficiency A sense of not being able to meet requirements (from work, university) Pressure to do as much as possible during isolation The “guilt and shame” spiral	Selective access to information Inaccurate information (including “fake news”) Prevalence of negative news stories	Limited access to grocery stores No access to clothing and industrial stores Lack of access to the service sector (e.g., hairdressers) Cancellation of events (e.g., weddings)	Having a family member or partner abroad Unable to return to Poland from abroad 2 weeks quarantine if returning from abroad Financial and housing problems Loneliness of foreign students Difficulty understanding new rules due to language barriers Stigmatization as bringers of the virus

Table 1 summarizes the range of challenges the students struggled with. We divided these challenges into three main categories: (1) mental health; (2) relationships; and (3) daily life. However, it is important to note that these categories are not mutually exclusive, and problems often “overlapped,” with students reporting symptoms from more than one category. The *Mental Health* category was divided into problems reported by the general population (almost all students) and problems reported by specific groups of students: those expecting a child, students with chronic diseases (diabetes and asthma, for example), and students struggling with addiction. The *Relationship* category contains problems that manifest in a student’s relationships with other groups. The COVID-19 pandemic negatively impacted relationships between students and their partners, children, other members of their families with whom they lived, and friends. Moreover, the pandemic also affected different aspects of *Daily life*. We divided problems in this category into six subcategories: (1) education (remote learning in particular); (2) work (mainly in relation to remote work and/or the possibility of job loss); (3) schedule (problems with effectively planning their day); (4) mass media and social media; (5) fulfilling needs (caused by limited access to many work, education, and social spaces); and (6) distance problems, which constituted a special group of difficulties in daily life. This pertained to both Polish people that were abroad and had difficulties returning to Poland as well as foreign students that faced different problems in Poland during the pandemic.

Symptoms of these problems are manifested as they would be in any stressful situation. However, they appear to be more intense in lockdown, as mass quarantine substantially raises levels of anxiety (Lima et al., 2020). A prominent recurring theme in many pandemic-related studies is the existential fear of being affected by COVID-19 and fear of death. Commonly reported symptoms include: fear, chronic stress, depressed mood, sleep problems (sleeplessness, waking up in the middle of the night, nightmares, poor sleep quality, and feeling fatigued during the day), intensification of symptoms of chronic diseases, and somatic symptoms (stomach pain, muscle aches, reproduction of virus symptoms, and hair loss). In extreme cases, suicidal thoughts occur, which can have severe consequences for one’s mental health and well-being (Bidzan et al., 2020; Bidzan-Bluma et al., 2020; Dymecka et al., 2021a,b; Schou-Bredal et al., 2021). Moreover, lockdowns have made it far more difficult to provide the psychological and emotional support required by those affected by such symptoms, which is why we sought the most effective ways to provide support through targeted interventions.

Since the first lockdown, we have observed that recurring difficulties continue to emerge. Although some people seem to be doing better after a year of experience (including through organizing online psychotherapy or having greater access to specialists than at the beginning of the pandemic), the consequences of the past 12months will stay with us for a long time.

## Applied Psychological Interventions

Our actions, especially at the beginning, were primarily based on a combination of professional experience and intuition. The pandemic situation was unprecedented, and none of us had a “ready-made” scenario or protocol to lean on. We were left to “go wild,” especially since there were no practical implementations of interventions at the time (Duan and Zhu, 2020). As time passed, however, official guidelines were released. We did not fully know what awaited us and what we would struggle with. At the very beginning of our service, we relied primarily on previously published guidelines for caring for mental health in isolation and pandemics as well as on our own experience, which is described below.

Fiorillo and Gorwood (2020) formulated five simple and universal pieces of advice for people in isolation, which were also used in our work. They relate to dimensions, such as (a) limiting sources of stress – including limiting time spent browsing information and referring only to reliable sources; (b) breaking the isolation through what contact with loved ones and friends was possible, such as *via* video-chat; (c) maintaining a consistent routine, including sleep and eating; (d) focusing on the positive effects of isolation – it helps us protect our health and not infect others; and (e) seeking professional help if needed. All of those were, to varying degrees, used in our practice.

We also based our interventions on the WHO recommendations published on the 18th March 2020 entitled: *Mental health and psychosocial considerations during the COVID-19 outbreak* (World Health Organization, 2020b). The WHO had advice for the general population, healthcare workers, team leaders (or managers in health facilities), carers of children and older adults, people in isolation, and people with underlying health conditions and their carers. We decided to use this document as a basic set of guidelines for our student support activities.

According to PMT, one crucial aspect of dealing with the consequences of the pandemic is maintaining one’s connection to oneself. This is possible through affective communication, self-reflection, enlargement of consciousness and feeling of wholeness, construction of life sense, and maintaining a biocentric lifestyle (Stueck, 2021). Together with the recommendations of the WHO, these guided our work and helped us to formulate the interventions below.

### Psychoeducation

Perceived lack of knowledge can often make a problem feel even more difficult to overcome. Therefore, we found it extremely useful to present and discuss the mechanisms underlying stress and anxiety or panic attacks in an accessible (and sometimes humorous) way. Scientific materials which effectively explained these difficult issues on social media channels, such as YouTube, proved to be a great help. Another effective tool we used was asking students to write self-narratives about the feelings and emotions they experienced. For some, this was the first time they had faced their problems and were able to name them, and, often, this was a sufficient platform from which to find a solution together. Structured letter therapy as a novel approach to consultations about COVID-19-related psychological and mental problems was proposed by Xiao (2020). This uses writing about “possible reasons for your current emotions, how you should ease them, or how to learn to live with them” as an intervention task. The author argues that this can play an important role when providing counselling for individuals experiencing psychological and mental problems that are prone to chronic progression, which is in line with our experience.

### Relaxation

Breathing exercises, relaxation music, yoga, and mindfulness training – although all these methods allow us to deal better with stress and anxiety, we tried to approach the issue of relaxation in a more personalized way by tailoring our interventions to the student’s way of life, temperament, interests, hobbies, and their use of free time. An effective method we used was sending students links to YouTube videos on various forms of relaxation, such as the Jacobson relaxation technique or yoga training for beginners. Mobile apps encouraging regular exercise and meditation also worked well. We additionally recommended relevant books on mindfulness, for example *The mindful way through depression*: *Freeing yourself from chronic unhappiness* (Williams et al., 2007), which is also available online in Polish. We also shared materials from programs similar to those prepared for our regular patients in hospitals, with some interesting activities based on positive psychology, such as practicing optimistic thinking or writing gratitude letters (Sin and Lyubomirsky, 2009; Yamaguchi et al., 2020). In special cases, we presented herbal tranquilizers available in pharmacies without a prescription, indicating that the pharmacist should be consulted regarding any contraindications.

### Arranging the Day Plan

It quickly became clear that one of the biggest challenges facing students during quarantine was how to effectively plan daily activities, including those connected with work and education (Rousseau and Miconi, 2020). The daily schedule also included how to healthily moderate media consumption, including newspapers and television news broadcasts. We recommended limiting this activity to twice a day and recommended accessing articles, videos, and materials from official sources, available online for free (in accordance with the WHO’s guidelines). Another important aspect of wellbeing discussed was how students could improve their sleep routines, for example by aiming for consistent sleeping and waking times (Hyun et al., 2021). Online studying also proved difficult for many students (and lecturers), especially at the start of lockdown, because online studying is not yet a widespread form of education in Poland. Planning tasks related to attending classes, passing courses, and preparing “homework” were often central to all other activities throughout the day, therefore we felt it was crucial to support students in continuing their education online to ensure as many students as possible completed their studies.

### Taking Care of Relationships

Relationships take on special significance in the context of isolation. For this reason, we emphasized the importance of maintaining contact over the phone or *via* instant messengers. We suggested that people might find it helpful to start a conversation with someone who feels lonely or with someone they had not talked to for a long time. This provides a good opportunity to renew old acquaintances and a good reason to finally have a chat. On the other hand, being forced to stay at home with someone we are afraid of is a different type of challenge. In such cases, we gave contact details for the appropriate institutions or organizations that help victims of domestic violence (e.g., crisis intervention centers).

### Long-Term Psychotherapy

Some students, especially those who had previously had a diagnosis of mental disorders (such as depression or obsessive-compulsive disorder) required long-term psychotherapy. We were able to give advice regarding where and how such therapy could be continued online during the quarantine. We collaborated with a group of experienced psychotherapists for this.

### “Just” a Simple Talk: The Art of Being and Listening

Sometimes “simply” being and listening is enough. For many people, it was priceless to be able to talk to someone about their fears and concerns. The feeling that on the other side there is a person who listens to us and at the same time devotes all his or her attention to us is very important.

The interventions used allowed us to provide direct help for both immediate interventions (e.g., expressing emotions, dealing with negative emotions, strengthening the motivation to seek help, looking for strengths to enhance situational coping, and cultivating healthy interpersonal relations and mental health) as well as long-term interventions (e.g., seeking a permanent form of therapy, rationalizing the pandemic situation, looking for ways to function effectively during the pandemic, developing effective daily planning skills, and enhanced relaxation techniques).

## Discussion: Challenges Faced and Reflections

We strongly believe that free psychological support for university students in this difficult time is necessary and will be in the next semester. The first stage of the pandemic, when none of us knew what the coming days would look like, showed this to be crucial. At the time of writing, we are still coping with challenges due to COVID-19 and trying to live with restrictions. It remains very difficult, but we are better prepared for and more understanding of the restrictions as well as the behavior of others. Nevertheless, the number of active cases and deaths in Poland is increasing every day. Being affected by COVID-19 carries additional adverse psychological consequences (Bidzan et al., 2020; Bidzan-Bluma et al., 2020; Khasawneh, 2020; Ranieri et al., 2020; Stamu-O’Brien et al., 2020; Super et al., 2020; Stueck, 2021) and the number of people who are affected is increasing – both in general and among UG students.

Our most notable observation was the fact that the people who contacted us were disproportionately female. Liddon et al. (2018) pointed out that men are less likely than women to seek treatment for mental health problems. COVID-19 tends to have a stronger psychological effect on women than it does on men, who display fewer signs of depression, anxiety, and post-traumatic stress disorder (PTSD), as well as feelings of isolation (Ausín et al., 2021; Yan et al., 2021). However, one should be careful here in formulating unequivocal conclusions, because cultural factors, among others, may play a large role here (Chatmon, 2020).

The problems reported by people who benefited from free support at the University of Gdańsk seem to be typical, as indicated by researchers from around the world (e.g., Mustafa, 2020). The most frequent challenges included anxiety, depression, stress, PTSD, and poor sleep quality. Both individual studies and meta-analyses have also confirmed that, globally, college students bear a disproportionate burden of mental health issues (Elmer et al., 2020; Wathelet et al., 2020; Batra et al., 2021; Bourion-Bédès et al., 2021). The support provided met some crucial criteria of the PMT (Stueck, 2021), for example, students at risk of loneliness were helped to maintain contact with others during the lockdown.

The problems and challenges faced by us were similar to those described by PMT (Stueck, 2021). At the beginning of the lockdown, most students appeared to be in the orientation phase, which quickly transitioned into an acute, chronic, and negatively loaded phase. Going forward, we may observe more students deriving more positive outcomes. We hope they have begun to deal with the challenges of the pandemic in more adaptive ways.

There is a certain element of selfishness in helping others, because being involved in such a project also helped us – psychologists – to feel needed and to feel satisfied and constructive in quarantine. However, this does not change the fact that we also faced our own anxieties and concerns – about our loved ones and ourselves. On one hand, we would like to assist anyone who comes to us, but, on the other, we still need to protect our own mental health. “A good lifeguard is a living lifeguard,” as we say in Poland – only if we take care of our own wellbeing first will we be able to help others (see PMT; Stueck, 2021).

Another feeling we experienced was the fear caused by the lack of clear guidelines and the simultaneous restricted access to specialists – including psychiatrists. This was particularly worrying when seeking help for students who had come to us as a “last resort.” We frequently experienced some kind of powerlessness when faced with students with financial problems and, similarly, students from abroad who felt “imprisoned” in Poland, unable to return to their countries. It can be very difficult indeed to experience isolation in a foreign country, away from home and loved ones. None of us can forecast the impact of the economic crisis caused by the COVID-19 pandemic; this is why it is so important to focus primarily on what we can actually influence.

We are not sure what kinds of psychological support will be needed in the future. However, researchers no longer have any doubts that the effects of the pandemic on university students will be long-term and therefore it is important to implement effective public health strategies to meet the emotional and psychosocial needs of college students (Elmer et al., 2020; Batra et al., 2021; Browning et al., 2021). We certainly need to monitor students’ mental health and promote mental health and well-being. The term “the psychological wound of COVID-19″ is already being used, and the effects of such wounds cannot be predicted (Padrón et al., 2021). The university is a unique setting that can play a key role in supporting young people by adapting methods of counselling to current needs, creating interactive therapeutic interventions (e.g., apps and online programs), and providing other services like text messages, chatlines, and forums (Sundarasen et al., 2020).

We, the specialists, as well as people who seek support from us, are aware that the world after the pandemic will not be the same. However, none of us can predict what changes will occur or what their consequences will be. *The only constant in life is change* as Heraclitus observed. On the other hand, we want to believe that this crisis will bring some secondary benefits, perhaps allowing us to appreciate what we previously took for granted. Currently, at least, we can see this in the statements of the people we have helped. Both psychological and psychotherapeutic interventions must, above all, be personalized and tailored to the needs of the individual. The reconstruction of meaning – making sense of COVID-19 – and interventions focused on individual and group resources with the aim of “normalizing” the current distressing situation seem to be the key issues for effective action (Castiglioni and Gaj, 2020; Inchausti et al., 2020; Milman et al., 2020; Venuleo et al., 2020).

## Strengths and Limitations

The main strength of the study is that it was conducted strictly after the COVID-19 pandemic had been announced in Poland. Thus, it was possible to document the creation of healthy identity from the beginning, but, more importantly, we were able to help people from the onset of this stressful situation. The help provided may also have positive consequences in the period after the pandemic, due to the self-reflection made possible by this challenging time and the strategies acquired for coping with the associated stress.

Furthermore, it was conducted in a non-artificial environment – at the workplace at the University of Gdańsk – with direct researcher–respondent contact (although *via* Skype).

The limitations of this study include a sample that is limited to students from only one university in the Pomeranian region of Poland, which makes it impossible to generalize the conclusions to students from other universities/countries. Another limitation of the study is the relatively small number of participants, which, however, is due to the fact that our non-experimental study is qualitative (narrative) rather than quantitative. Furthermore, the data collected were from a single, fixed period (between the 18th March and 15th June 2020) and is not longitudinal. We were unable to include all variables which could affect mental health, relationships, socioeconomic status, and the daily life of student populations during the COVID-19 pandemic due to limited time and resources.

## Conclusion

There is a great need to provide adequate, personalized, and evidence-based psychological support to student populations. The pandemic will likely have long-term implications, and as it continues to unfold, new challenges will appear. Hence it is important to share best practices as well as lessons learned from difficulties encountered, to find optimal solutions together moving forward (World Health Organization, 2021). We are witnessing a change in the challenges faced by individuals experiencing psychological problems, such as depressive and anxiety disorders, and increased feelings of hopelessness and frustration. Now, exactly 1year after the first case of SARS-COV-2 was detected in Poland, the level of anxiety among the inhabitants of this country is still very high. The statistics do not inspire optimism. As of the 5th April 2021, 2.44 million people in Poland have been infected, with 54,941 recorded deaths (WHO). We all look with hope to the progressing vaccination process (on the 2nd April, 12% of the population had been vaccinated with at least one dose and 5.5% with two doses (COVID-19 Data, 2021). We hope and believe that we will return to the university campus in the next academic year. Now, as we finish our work on this article, Poland has been hit by a third wave of the COVID-19 pandemic. The numbers of people infected and dying each day have never been so high. We see our students getting tired of e-learning and experiencing frustration. Many of them have struggled or are still struggling with health issues, lost their loved ones, and are fighting for economic survival. At the very beginning, we asked “would we organize our psychological service to meet this challenge?” The answer was yes. We used all the resources that were available to us. The most important thing for us was to act! Each of us gave our best, as we saw how quickly the mental health of the students began to deteriorate. In retrospect, what seemed to be the greatest challenge to us were the formal and technical issues: on one hand, the need to operate ethically, for example by ensuring anonymity, and on the other, the need to provide support to students as rapidly as possible. It is also important to note that it was difficult for us to take care of our own mental health and maintain a healthy work–life balance. We have no doubts that the need for psychological help will continue to increase, so it is important to publish materials that can provide knowledge and inspiration for healthcare professionals undertaking similar psychological help initiatives.

## Epilogue

We can now, with unabashed pride and satisfaction, share the news that the success of our psychological support initiative has since led to the establishment of an Academic Psychological Support Center at the University of Gdańsk. From March 2021, both undergraduates and postgraduates, as well as employees (research, teaching, and administrative), can benefit from free psychological support. Currently, due to the ongoing epidemiological threat, it is being provided online (using Skype and Microsoft Teams). Depending on personal needs, consultations require an appointment or can be carried out immediately. Individuals have access to three sessions, which may last from 30 to 50min. In total, six psychologists are involved in the work of the Center, all of whom have experience in psychological counselling (including at least 3years of work in the field of clinical psychology) or are active psychotherapists. A total of 12h of meetings are held every week. The need for this initiative is evidenced by the fact that we are currently booked up 1month in advance, and more people visit us every day. The Psychological Support Center has already undertaken grants aimed at improving the well-being of the academic community and youth from secondary schools. We plan to conduct research projects to better understand and minimize the psychological effects of the pandemic.

## Data Availability Statement

The original contributions presented in the study are included in the article/supplementary material, further inquiries can be directed to the corresponding author.

## Ethics Statement

The studies involving human participants were reviewed and approved by the Ethical Committee (decision no. 30/2020) at the Institute of Psychology at the University of Gdańsk, Poland. Written informed consent for participation was not required for this study in accordance with the national legislation and the institutional requirements.

## Author Contributions

AR: the general idea of publication, experience sharing, and article editing. PA-W and AS-W: experience sharing and article editing. PC: article editing and linguistic proofreading. MB: supervision. All authors contributed to the article and approved the submitted version.

## Conflict of Interest

The authors declare that the research was conducted in the absence of any commercial or financial relationships that could be construed as a potential conflict of interest.

## Publisher’s Note

All claims expressed in this article are solely those of the authors and do not necessarily represent those of their affiliated organizations, or those of the publisher, the editors and the reviewers. Any product that may be evaluated in this article, or claim that may be made by its manufacturer, is not guaranteed or endorsed by the publisher.

## Footnotes

^1^As of completion of this article, the above regulation was still in force along with updated regulations of ordinances No. 39 / R / 20 and No. 48/R/21 of the Rector of the University of Gdańsk.

## References

[ref1] Ammar A. Brach M. Trabelsi K. Chtourou H. Boukhris O. Masmoudi L.. (2020). Effects of COVID-19 home confinement on eating behaviour and physical activity: results of the ECLB-COVID19 international online survey. Nutrients 12:1583. doi: 10.3390/nu12061583, PMID: 32481594 PMC7352706

[ref2] Ausín B. González-Sanguino C. Castellanos M. Á. Muñoz M. (2021). Gender-related differences in the psychological impact of confinement as a consequence of COVID-19 in Spain. J. Gend. Stud. 30, 29–38. doi: 10.1080/09589236.2020.1799768

[ref3] Bartoszek A. Walkowiak D. Bartoszek A. Kardas G. (2020). Mental well-being (depression, loneliness, insomnia, daily life fatigue) during COVID-19 related home-confinement—A study from Poland. Int. J. Environ. Res. Public Health 17:7417. doi: 10.3390/ijerph17207417, PMID: 33053787 PMC7599953

[ref4] Batra K. Sharma M. Batra R. Singh T. Schvaneveldt N. (2021). Assessing the psychological impact of COVID-19 among college students: an evidence of 15 countries. Health 9:222. doi: 10.3390/healthcare9020222, PMID: 33671363 PMC7923198

[ref5] Bidzan M. Bidzan-Bluma I. Szulman-Wardal A. Stueck M. Bidzan M. (2020). Does self-efficacy and emotional control protect hospital staff from COVID-19 anxiety and PTSD aymptoms? Psychological functioning of hospital staff after the announcement of COVID-19 coronavirus pandemic. Front. Psychol. 11:552583. doi: 10.3389/fpsyg.2020.552583, PMID: 33424673 PMC7785971

[ref6] Bidzan-Bluma I. Bidzan M. Jurek P. Bidzan L. Knietzsch J. Stueck M.. (2020). A polish and German population study of quality of life, well-being, and life satisfaction in older adults during the COVID-19 pandemic. Front. Psychol. 11:585813. doi: 10.3389/fpsyt.2020.585813, PMID: 33281646 PMC7705096

[ref7] Bourion-Bédès S. Tarquinio C. Batt M. Tarquinio P. Lebreuilly R. Sorsana C.. (2021). Psychological impact of the COVID-19 outbreak on students in a French region severely affected by the disease: results of the PIMS-CoV 19 study. Psychiatry Res. 295:113559. doi: 10.1016/j.psychres.2020.113559, PMID: 33189368 PMC7644189

[ref8] Brooks S. K. Webster R. K. Smith L. E. Woodland L. Wessely S. Greenberg N. (2020). The psychological impact of quarantine and how to reduce it: rapid review of the evidence. Lancet 395, P912–P920. doi: 10.1016/S0140-6736(20)30460-8, PMID: 32112714 PMC7158942

[ref9] Browning M. H. E. M. Larson L. R. Sharaievska I. Rigolon A. McAnirlin O. Mullenbach L.. (2021). Psychological impacts from COVID-19 among university students: risk factors across seven states in the United States. PLoS One 16:e0245327. doi: 10.1371/journal.pone.0245327, PMID: 33411812 PMC7790395

[ref10] Cao W. Fang Z. Hou G. Han M. Xu X. Dong J.. (2020). The psychological impact of the COVID-19 epidemic on college students in China. Psychiatry Res. 287:112934. doi: 10.1016/j.psychres.2020.112934, PMID: 32229390 PMC7102633

[ref11] Castiglioni M. Gaj N. (2020). Fostering the reconstruction of meaning among the general population during the COVID-19 pandemic. Front. Psychol. 11:567419. doi: 10.3389/fpsyg.2020.567419, PMID: 33192849 PMC7655933

[ref12] Chatmon B. N. (2020). Males and mental health stigma. Am. J. Mens Health 14:1557988320949322. doi: 10.1177/1557988320949322, PMID: 32812501 PMC7444121

[ref13] COVID-19 Data (2021). Available at: https://github.com/owid/covid-19-data/blob/master/public/data/vaccinations/country_data/Poland.csv (Accessed April 5, 2021).

[ref14] Duan L. Zhu G. (2020). Psychological interventions for people affected by the COVID-19 epidemic. Lancet Psychiatry 7, 300–302. doi: 10.1016/S2215-0366(20)30073-0, PMID: 32085840 PMC7128328

[ref15] Dymecka J. Gerymski R. Machnik-Czerwik A. (2021a). How does stress affect our life satisfaction during COVID-19 pandemic? Moderating mediation analysis of sense of coherence and fear of coronavirus. Psychol. Health Med.. doi: 10.1080/13548506.2021.1906436 [Epub ahead of print] 33784897

[ref66] Dymecka J. Gerymski R. Machnik-Czerwik A. (2021b). Fear of COVID-19 as a buffer in the relationship between perceived stress and life satisfaction in the Polish population at the beginning of the global pandemic. Health Psychol. Rep. 9, 149–159. doi: 10.5114/hpr.2020.102136 PMC10501413 38084284

[ref16] Elmer T. Mepham K. Stadtfeld C. (2020). Students under lockdown: comparisons of students’ social networks and mental health before and during the COVID-19 crisis in Switzerland. PLoS One 15:e0236337. doi: 10.1371/journal.pone.0236337, PMID: 32702065 PMC7377438

[ref17] Fiorenzato E. Zabberoni S. Costa A. Cona G. (2021). Cognitive and mental health changes and their vulnerability factors related to COVID-19 lockdown in Italy. PLoS One 16:e0246204. doi: 10.1371/journal.pone.0246204, PMID: 33503055 PMC7840042

[ref18] Fiorillo A. Gorwood P. (2020). The consequences of the COVID-19 pandemic on mental health and implications for clinical practice. Eur. Psychiatry 63:e32. doi: 10.1192/j.eurpsy.2020.35, PMID: 32234102 PMC7156565

[ref67] Fruehwirth J. C. Biswas S. Perreira K. M. (2021). The Covid-19 pandemic and mental health of first-year college students: examining the effect of Covid-19 stressors using longitudinal data. PLoS One 16:e0247999. doi: 10.1371/journal.pone.0247999 33667243 PMC7935268

[ref19] Gambin M. Sękowski M. Woźniak-Prus M. Wnuk A. Oleksy T. Cudo A.. (2021). Generalized anxiety and depressive symptoms in various age groups during the COVID-19 lockdown in Poland. Specific predictors and differences in symptoms severity. Compr. Psychiatry 105:152222. doi: 10.1016/j.comppsych.2020.152222, PMID: 33388494

[ref20] Gawrych M. Cichoń E. Kiejna A. (2020). COVID-19 pandemic fear, life satisfaction and mental health at the initial stage of the pandemic in the largest cities in Poland. Psychol. Health Med. 26, 107–113. doi: 10.1080/13548506.2020.1861314, PMID: 33300378

[ref21] Grubic N. Badovinac S. Johri A. M. (2020). Student mental health in the midst of the COVID-19 pandemic: a call for further research and immediate solutions. Int. J. Soc. Psychiatry 66, 517–518. doi: 10.1177/0020764020925108, PMID: 32364039 PMC7405631

[ref22] Hyun S. Hahm H. C. Wong G. T. F. Zhang E. Liu C. H. (2021). Psychological correlates of poor sleep quality among US young adults during the COVID-19 pandemic. Sleep Med. 78, 51–56. doi: 10.1016/j.sleep.2020.12.009, PMID: 33385779 PMC7887075

[ref23] Inchausti F. MacBeth A. Hasson-Ohayon I. Dimaggio G. (2020). Psychological intervention and COVID-19: what we know so far and what we can do. J. Contemp. Psychother. 50, 243–250. doi: 10.1007/s10879-020-09460-w, PMID: 32836375 PMC7250659

[ref24] Khasawneh M. (2020). The effect of the spread of the new COVID-19 on the psychological and social adaptation of families of persons with disabilities in the Kingdom of Saudi Arabia. Health Psychol. Rep. 9, 264–275. doi: 10.5114/hpr.2020.99003 PMC10694694 38084228

[ref25] Liddon L. Kingerlee R. Barry J. A. (2018). Gender differences in preferences for psychological treatment, coping strategies, and triggers to help-seeking. Br. J. Clin. Psychol. 57, 42–58. doi: 10.1111/bjc.12147, PMID: 28691375

[ref26] Lima C. K. T. de Medeiros Carvalho P. M. Lima I. D. A. S. de Oliveira Nunes J. V. A. Saraiva J. S. de Souza R. I.. (2020). The emotional impact of coronavirus 2019-nCoV (new coronavirus disease). Psychiatry Res. 287:112915. doi: 10.1016/j.psychres.2020.112915, PMID: 32199182 PMC7195292

[ref27] Liu C. H. Pinder-Amaker S. Hahm H. C. Chen J. A. (2020). Priorities for addressing the impact of the COVID-19 pandemic on college student mental health. J. Am. Coll. Heal. 1–3. doi: 10.1080/07448481.2020.1803882 [Epub ahead of print] PMC8041897 33048654

[ref28] Milman E. Lee S. A. Neimeyer R. A. (2020). Social isolation and the mitigation of coronavirus anxiety: the mediating role of meaning. Death Stud. 1–13. doi: 10.1080/07481187.2020.1775362 [Epub ahead of print] 32544375

[ref29] Mustafa A. K. (2020). Psychological consequences of COVID-19 and challenges for post-traumatic interventions. J. Psychol. Res. 10, 24–29. doi: 10.17265/2159-5542/2020.01.003

[ref30] Padrón I. Fraga I. Vieitez L. Montes C. Romero E. (2021). A study on the psychological wound of COVID-19 in university students. Front. Psychol. 12:589927. doi: 10.3389/fpsyg.2021.589927, PMID: 33574786 PMC7870473

[ref31] Patsali M. E. Mousa D. P. V. Papadopoulou E. V. Papadopoulou K. K. Kaparounaki C. K. Diakogiannis I.. (2020). University students’ changes in mental health status and determinants of behavior during the COVID-19 lockdown in Greece. Psychiatry Res. 292:113298. doi: 10.1016/j.psychres.2020.113298, PMID: 32717710 PMC7357537

[ref32] Pedrosa A. L. Bitencourt L. Fróes A. Cazumbá M. Campos R. de Brito S.. (2020). Emotional, behavioral, and psychological impact of the COVID-19 pandemic. Front. Psychol. 11:566212. doi: 10.3389/fpsyg.2020.566212, PMID: 33117234 PMC7561666

[ref33] Pfefferbaum B. North C. S. (2020). Mental health and the Covid-19 pandemic. N. Engl. J. Med. 383, 510–512. doi: 10.1056/NEJMp2008017, PMID: 32283003

[ref34] Polish Press Agency (2020). Available at: https://naukawpolsce.pap.pl/aktualnosci/news%2C81262%2Cgdansk-w-zwiazku-z-koronawirusem-ug-oferuje-pomoc-psychologiczna-online.html (Accessed April 18, 2020).

[ref35] Qiu J. Shen B. Zhao M. Wang Z. Xie B. Xu Y. (2020). A nationwide survey of psychological distress among Chinese people in the COVID-19 epidemic: implications and policy recommendations. General Psychiatry 33:e100213. doi: 10.1136/gpsych-2020-100213, PMID: 32215365 PMC7061893

[ref36] Ranieri J. Guerra F. Giacomo D. (2020). Predictive risk factors for post-traumatic stress symptoms among nurses during the Italian acute COVID-19 outbreak. Health Psychol. Rep. 9, 180–185. doi: 10.5114/hpr.2020.101249 PMC10501412 38084281

[ref37] Republic of Poland – website (2020). Available at: https://www.gov.pl/web/koronawirus/wprowadzamy-stan-epidemii-w-polsce (Accessed April 10, 2020).

[ref38] Rossi R. Socci V. Talevi D. Mensi S. Niolu C. Pacitti F.. (2020). COVID-19 pandemic and lockdown measures impact on mental health among the general population in Italy. Front. Psychol. 11:790. doi: 10.3389/fpsyt.2020.00790, PMID: 32848952 PMC7426501

[ref39] Rousseau C. Miconi D. (2020). Protecting youth mental health during the COVID-19 pandemic: A challenging engagement and learning process. J. Am. Acad. Child Adolesc. Psychiatry 59, 1203–1207. doi: 10.1016/j.jaac.2020.08.007, PMID: 33126993

[ref40] Schou-Bredal I. Grimholt T. Bonsaksen T. Skogstad L. Heir T. Ekeberg Ø. (2021). Optimists’ and pessimists’ self-reported mental and global health during the COVID-19 pandemic in Norway. Health Psychol.Rep. 9, 160–168. doi: 10.5114/hpr.2021.102394 PMC10501414 38084286

[ref41] Sin N. L. Lyubomirsky S. (2009). Enhancing well-being and alleviating depressive symptoms with positive psychology interventions: a practice-friendly meta-analysis. J. Clin. Psychol. 65, 467–487. doi: 10.1002/jclp.20593, PMID: 19301241

[ref42] Son C. Hegde S. Smith A. Wang X. Sasangohar F. (2020). Effects of COVID-19 on college students’ mental health in the United States: interview survey study. J. Med. Internet Res. 22:e21279. doi: 10.2196/21279, PMID: 32805704 PMC7473764

[ref43] Stamu-O’Brien C. Carniciu S. Halvorsen E. Jafferany M. (2020). Psychological aspects of COVID-19. J. Cosmet. Dermatol. 19, 2169–2173. doi: 10.1111/jocd.13601, PMID: 33439544

[ref44] Stueck M. (2021). The pandemic management theory. COVID-19 and biocentric development. Health Psychol.Rep. 9, 101–128. doi: 10.5114/hpr.2021.103123 PMC10687539 38084288

[ref45] Sundarasen S. Chinna K. Kamaludin K. Nurunnabi M. Baloch G. M. Khoshaim H. B.. (2020). Psychological impact of COVID-19 and lockdown among university students in Malaysia: implications and policy recommendations. Int. J. Environ. Res. Public Health 17:6206. doi: 10.3390/ijerph17176206, PMID: 32867024 PMC7504527

[ref46] Super S. Pijpker R. Polhuis K. (2020). The relationship between individual, social and national coping resources and mental health during the COVID-19 pandemic in the Netherlands. Health Psychol. Rep. 9, 186–192. doi: 10.5114/hpr.2020.99028 PMC10687525 38084283

[ref48] Townsend E. Nielsen E. Allister R. Cassidy S. A. (2020). Key ethical questions for research during the COVID-19 pandemic. Lancet Psychiatry 7, 381–383. doi: 10.1016/S2215-0366(20)30150-4, PMID: 32353264 PMC7185919

[ref49] University of Gdańsk (2020a). Regulation No. 20/R/20 of the Rector of the University of Gdańsk of March 10 2020 on the prevention of the spread of the SARS-Cov-2 coronavirus.

[ref50] University of Gdańsk (2020b). Available at: https://ug.edu.pl/news/en/227/free-line-psychological-support-university-gdansk (Accessed April 10, 2020).

[ref51] University of Gdańsk (2020c). Available at: https://www.facebook.com/UniwersytetGdanski/ (Accessed April 10, 2020).

[ref52] University of Gdańsk (2021). Regulation No. 48/R/20 of the Rector of the University of Gdańsk of March 19 2021 on the prevention of the spread of the SARS-Cov-2 coronavirus.

[ref53] Venuleo C. Gelo C. G. O. Salvatore S. (2020). Fear, affective semiosis, and management of the pandemic crisis: COVID-19 as semiotic vaccine? Clin. Neuropsychiatry 17, 117–130. doi: 10.36131/CN20200218 PMC8629038 34908982

[ref54] Visible Hand 3City (2020). Available at: https://www.facebook.com/groups/WR3MIASTO/ (Accessed April, 10 2020).

[ref55] Wang X. Hegde S. Son C. Keller B. Smith A. Sasangohar F. (2020a). Investigating mental health of US college students during the COVID-19 pandemic: cross-sectional survey study. J. Med. Internet Res. 22:e22817. doi: 10.2196/22817, PMID: 32897868 PMC7505693

[ref56] Wang C. Pan R. Wan X. Tan Y. Xu L. Ho C. S.. (2020b). Immediate psychological responses and associated factors during the initial stage of the 2019 coronavirus disease (COVID-19) epidemic among the general population in China. Int. J. Environ. Res. Public Health 17:1729. doi: 10.3390/ijerph17051729, PMID: 32155789 PMC7084952

[ref57] Wang Y. Shi L. Que J. Lu Q. Liu L. Lu Z.. (2021). The impact of quarantine on mental health status among general population in China during the COVID-19 pandemic. Mol. Psychiatry doi: 10.1038/s41380-021-01019-y [Epub ahead of print] PMC7821451 33483692

[ref58] Wathelet M. Duhem S. Vaiva G. Baubet T. Habran E. Veerapa E.. (2020). Factors associated with mental health disorders among university students in France confined during the COVID-19 pandemic. JAMA Netw. Open 3:e2025591. doi: 10.1001/jamanetworkopen.2020.25591, PMID: 33095252 PMC7584927

[ref47] Williams J. M. G. Teasdale J. Segal Z. Kabat-Zinn J. (2007). The Mindful Way through Depression: Freeing yourself from Chronic Unhappiness. New York and London: Guilford Press.

[ref59] World Health Organization (2020a). Coronavirus disease 2019 (COVID-19) – Situation Report – 50. WHO. 10 March 2020. Archived from the original on 10 April 2020. Available at: https://www.who.int/docs/default-source/coronaviruse/situation-reports/20200310-sitrep-50-covid-19.pdf (Accessed April 10, 2020).

[ref60] World Health Organization (2020b). Mental health and psychosocial considerations during the COVID-19 outbreak, 18 March 2020 (No. WHO/2019-nCoV/MentalHealth/2020.1). World Health Organization. Available at: https://www.who.int/docs/default-source/coronaviruse/mental-health-considerations.pdf (Accessed April 10, 2020).

[ref61] World Health Organization (2021). Available at: https://covid19.who.int/region/euro/country/pl (Accessed April 5, 2021).

[ref62] Xiao C. (2020). A novel approach of consultation on 2019 novel coronavirus (COVID-19)-related psychological and mental problems: structured letter therapy. Psychiatry Investig. 17, 175–176. doi: 10.30773/pi.2020.0047, PMID: 32093461 PMC7047000

[ref63] Yamaguchi K. Takebayashi Y. Miyamae M. Komazawa A. Yokoyama C. Ito M. (2020). Role of focusing on the positive side during COVID-19 outbreak: mental health perspective from positive psychology. Psychol. Trauma Theory Res. Pract. Policy 12, S49–S50. doi: 10.1037/tra0000807, PMID: 32525365

[ref64] Yan S. Xu R. Stratton T. D. Kavcic V. Luo D. Hou F.. (2021). Sex differences and psychological stress: responses to the COVID-19 pandemic in China. BMC Public Health 21:79. doi: 10.1186/s12889-020-10085-w, PMID: 33413224 PMC7789895

[ref65] Zhao X. Fan J. Basnyat I. Hu B. (2020). Online health information seeking using “# COVID-19 patient seeking help” on Weibo in Wuhan, China: descriptive study. J. Med. Internet Res. 22:e22910. doi: 10.2196/22910, PMID: 33001838 PMC7572118

